# Crosstalk between osteoprotegerin (OPG), fatty acid synthase (FASN) and, cycloxygenase-2 (COX-2) in breast cancer: implications in carcinogenesis

**DOI:** 10.18632/oncotarget.9835

**Published:** 2016-06-06

**Authors:** Sudeshna Goswami, Neelam Sharma-Walia

**Affiliations:** ^1^ H. M. Bligh Cancer Research Laboratories, Department of Microbiology and Immunology, Chicago Medical School, Rosalind Franklin University of Medicine and Science, North Chicago, Illinois, U.S.A.

**Keywords:** CRISPR/Cas9, osteoprotegerin, cyclooxygenase-2, fatty acid synthase, breast cancer

## Abstract

The crosstalk between malignant and nonmalignant cells in the tumor microenvironment, as maneuvered by cytokines/chemokines, drives breast cancer progression. In our previous study, we discovered Osteoprotegerin (OPG) as one of the cytokines heavily secreted by breast cancer cells. We demonstrated that OPG is expressed and secreted at very high levels from the highly invasive breast cancer cell lines SUM149PT and SUM1315MO2 as compared to normal human mammary epithelial HMEC cells. OPG was involved in modulating aneuploidy, cell proliferation, and angiogenesis in breast cancer. Mass spectrometry analysis performed in this study revealed OPG interacts with fatty acid synthase (FASN), which is a key enzyme of the fatty acid biosynthetic pathway in breast cancer cells. Further, electron microscopy, immunofluorescence, and fluorescence quantitation assays highlighted the presence of a large number of lipid bodies (lipid droplets) in SUM149PT and SUM1315MO2 cells in comparison to HMEC. We recently showed upregulation of the COX-2 inflammatory pathway and its metabolite PGE2 secretion in SUM149PT and SUM1315MO2 breast cancer cells. Interestingly, human breast cancer tissue samples displayed high expression of OPG, PGE2 and fatty acid synthase (FASN). FASN is a multifunctional enzyme involved in lipid biosynthesis. Immunofluorescence staining revealed the co-existence of COX-2 and FASN in the lipid bodies of breast cancer cells. We reasoned that there might be crosstalk between OPG, FASN, and COX-2 that sustains the inflammatory pathways in breast cancer. Interestingly, knocking down OPG by CRISPR/Cas9 gene editing in breast cancer cells decreased FASN expression at the protein level. Here, we identified cis-acting elements involved in the transcriptional regulation of COX-2 and FASN by recombinant human OPG (rhOPG). Treatment with FASN inhibitor C75 and COX-2 inhibitor celecoxib individually decreased the number of lipid bodies/cell, downregulated phosphorylation of ERK, GSK3β, and induced apoptosis by caspase-3/7 and caspase-9 activation. But a more efficient and effective decrease in lipid bodies/cell and survival kinase signaling was observed upon combining the drug treatments for the aggressive cancer cells. Collectively, the novel biological crosstalk between OPG, FASN, and COX-2 advocates for combinatorial drug treatment to block these players of carcinogenesis as a promising therapeutic target to treat highly invasive breast cancer.

## INTRODUCTION

Breast cancer is the most common cancer and the second leading cause of death from malignancy in women in the United States. Highly metastatic inflammatory breast cancer (IBC) is a rare and lethal form of breast cancer affecting roughly 1-6% of all breast cancer patients. IBC is treated using a multimodal approach but patients have a poor prognosis, and have a high mortality rate, due to the ineffective and toxic chemotherapy [1]. Thus, there is an urgent need for safe and efficacious drugs that can combat this aggressive breast cancer.

OPG (osteoprotegerin) is a secreted member of the TNFR (tumor necrosis factor receptor) superfamily of proteins. It is a secretory protein and, as evident from the name, it blocks the maturation of bone resorbing osteoclast cells in the bone microenvironment thus preventing bone resorption [2]. Our previous study [3], for the first time, identified that OPG is secreted and expressed at very high levels from SUM1315MO2 (invasive breast cancer cell line), SUM149PT and SUM190PT cells (inflammatory breast cancer cell lines). OPG could reprogram the normal HMEC cells by inducing cell survival, proliferation, aneuploidy and upregulating CD24 expression. OPG also induced upregulation in gene copy numbers for oncogenic pathway regulators such Aurora A, EGFR, AKT/PI3K, and MYC in HMEC spheres [3].

Lipid bodies, with their associated elevated progenesis, have emerged as dynamic organelles involved in lipid metabolism and inflammation. Increased lipid body numbers have been observed in various kinds of tumor cells, and are recognized as potential targets for therapeutic interventions [4, 5]. Increased lipogenesis has been associated with poor prognosis in breast, prostate, and colon cancer [4, 5]. Fatty acid synthesis, the process of producing de novo fatty acids from carbohydrate and amino acid-derived carbon sources, is controlled by the enzyme fatty acid synthase (FASN) [6]. It is a multifunctional polypeptide enzyme that produces saturated fatty acids, uses one acetyl-CoA and sequentially adds seven malonyl-CoA molecules to produce the saturated 16-carbon palmitic acid [6]. Overexpression of FASN has been strongly associated with many cancer types and is under extensive study as a potential cancer drug target [6]. FASN plays an important metabolic role in molecular pathways regulating cancer cell proliferation and tumor development. Reduction in FASN enzyme activity by chemical inhibitors including orlistat, cerulenin (unapproved for use in humans) and triclosan have been reported to remarkably decrease progression in various cancer cell types [7]. There is a strong link between FASN and the cyclooxygenase (COX) pathway in cancer. Upregulation of FASN expression in cancer has been shown to be consistent with metabolism of arachidonic acid by the COX pathway, a crucial player in inflammation and cancer progression [8]. Also, downregulation of FASN in cancer has contributed to chemopreventive action of celecoxib, a drug for the COX-2 pathway [9].

Levels of COX-2 are tightly controlled in most tissues, and its gene regulation is exclusively dependent on gene transcription and posttranscriptional events [10]. COX-2 promoter regions from human [11], mouse [12], rat [13, 14], and chicken [15] have been cloned and their expression is tightly regulated at both the transcriptional and post-transcriptional levels. COX-2 is overexpressed in human cancers such as lung, breast, stomach, prostate, ovarian, head and neck, pancreatic, brain, glioma, melanoma, colorectal adenoma and adenocarcinoma [16]. COX-2 is the enzyme that catalyzes the rate limiting step in eicosanoid synthesis, converting arachidonic acid into prostaglandins [17]. PGE2, one of the main products of COX-2, is considered to be the prostaglandin mainly responsible for the pro-oncogenic action of COX-2, likely through the activation of the EP receptors, causing the activation of multiple tyrosine kinases [16]. PGE2 inhibits apoptosis, promotes angiogenesis, proliferation and metastasis [16]. PGE2 can induce a number of pro-inflammatory mediators in form of chemokines and even COX-2, which, in turn, promote cell proliferation and survival, angiogenesis, invasion, and metastasis [16]. Interestingly, COXIBs appear to be effective in treating adenocarcinomas in patients with familiar adenomatous polyposis [16].

In this study, we investigated the anti-tumorigenic, anti-lipogenic, and anti-inflammatory potential of COX-2 inhibitor celecoxib and FASN blocker C75 on two specific breast cancer cell lines. Our results suggest that celecoxib and C75 could act in a concerted way with improved therapeutic potential in invasive breast cancer.

## RESULTS

### Breast cancer cells contain lipid bodies and express high levels of FASN

**Figure 1:**
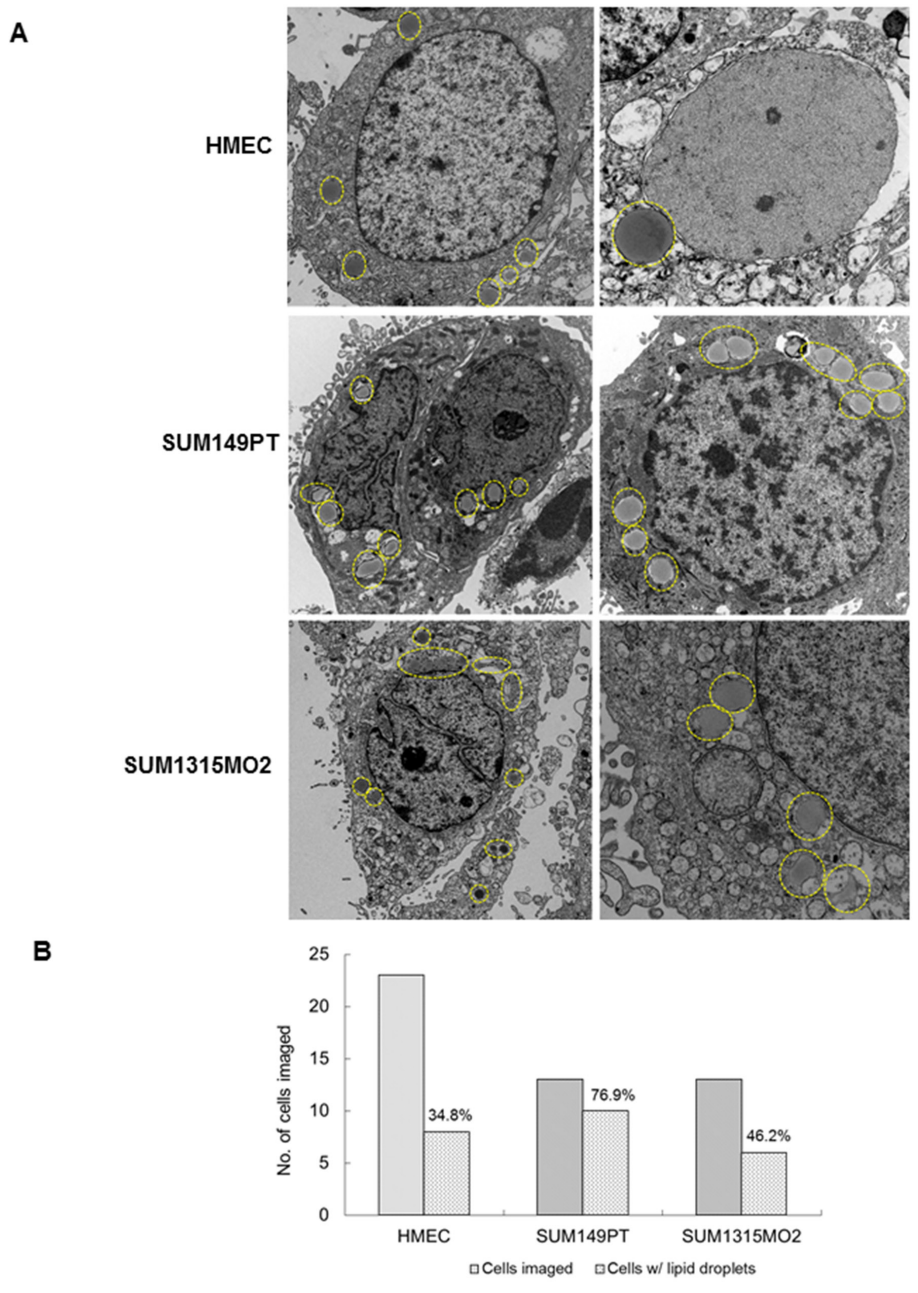
Electron microscopy of breast cancer cells **A.** Electron microscopy images of HMEC, SUM149PT and SUM1315MO2 cells. The cells were washed, fixed, processed for transmission EM, and embedded in 812 resin. Thin sections were made and visualized under a JEOL 100CXII transmission electron microscope. Results shown are representative images of three different fields and EM analysis was performed in triplicate. **B.** Quantification of the lipid bodies as previously observed in the electron microscopy images of HMEC, SUM149PT and SUM1315MO2 cells.

**Figure 2:**
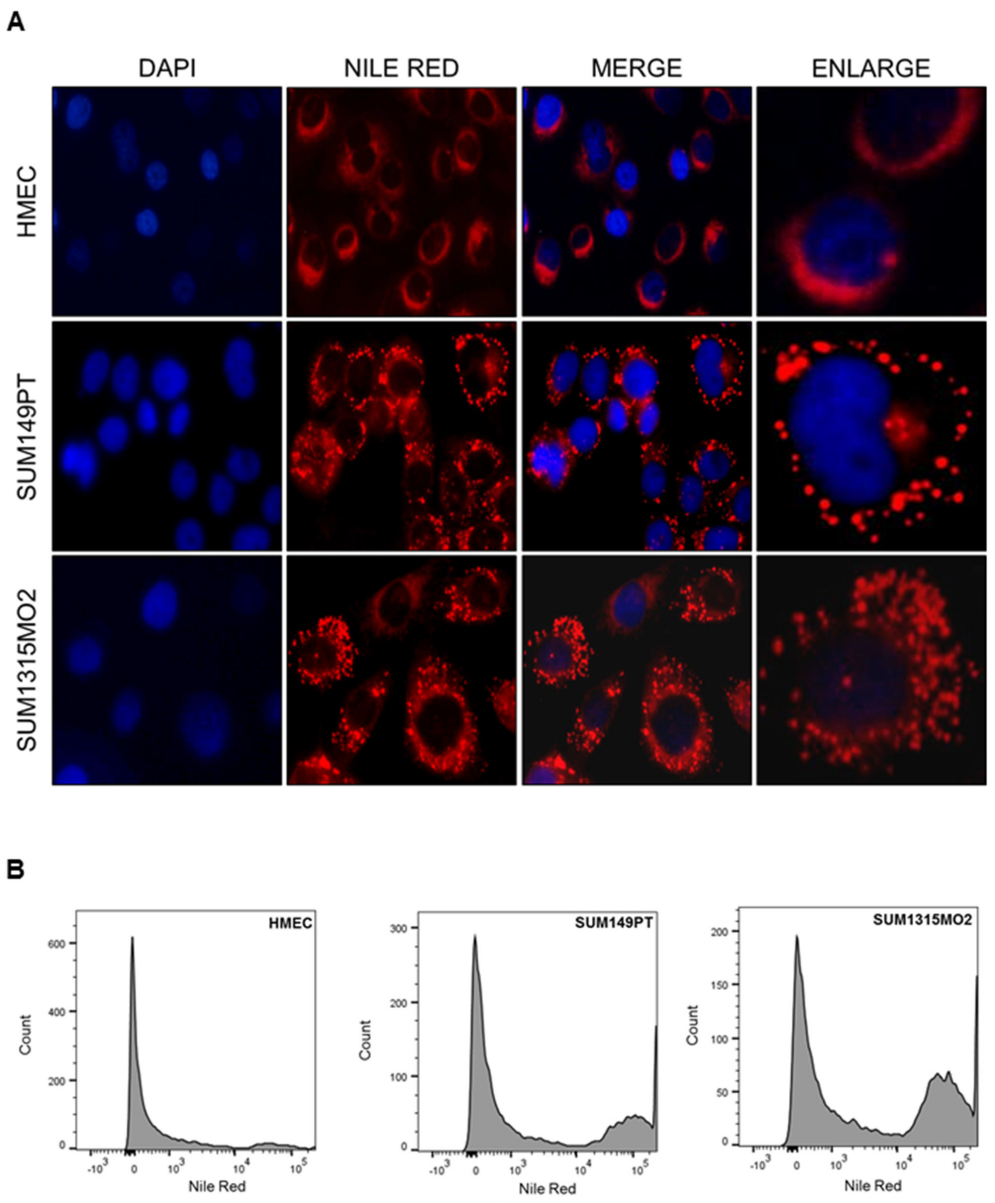
Breast cancer cells express high lipid body content **A.** Cells were fixed and immunofluorescence analysis was done using Nile red to stain lipid bodies and counter stained with DAPI. Magnification for the panels is 20X. Red dots indicate lipid bodies. Scale is 20μm. **B.** Flow cytometry analysis of the Nile red stained lipid bodies in breast cancer cells in comparison to HMEC. After washing, cells were fixed, incubated with Nile red and examined by FACS.

Electron microscopy (EM) imaging was performed to visualize lipid bodies in HMEC, SUM149PT and SUM1315MO2 cells (Figure 1A). The control HMEC cells had very few small lipid bodies (Figure 1A) while, SUM149PT and SUM1315MO2 cells had a larger number of bigger and denser lipid bodies (Figure 1A). Quantitatively, 76.9% of SUM149PT and 46.2% of SUM1315MO2 cells imaged had lipid bodies in comparison to 34.8% of HMEC cells (Figure 1B). Staining with Nile red further confirmed the abundance of lipid bodies in SUM149PT and SUM1315MO2 breast cancer cells (Figure 2A). Flow cytometry revealed an overabundance of lipid bodies in SUM149PT and SUM1315MO2 cells when compared to HMEC (Figure 2B).

**Figure 3:**
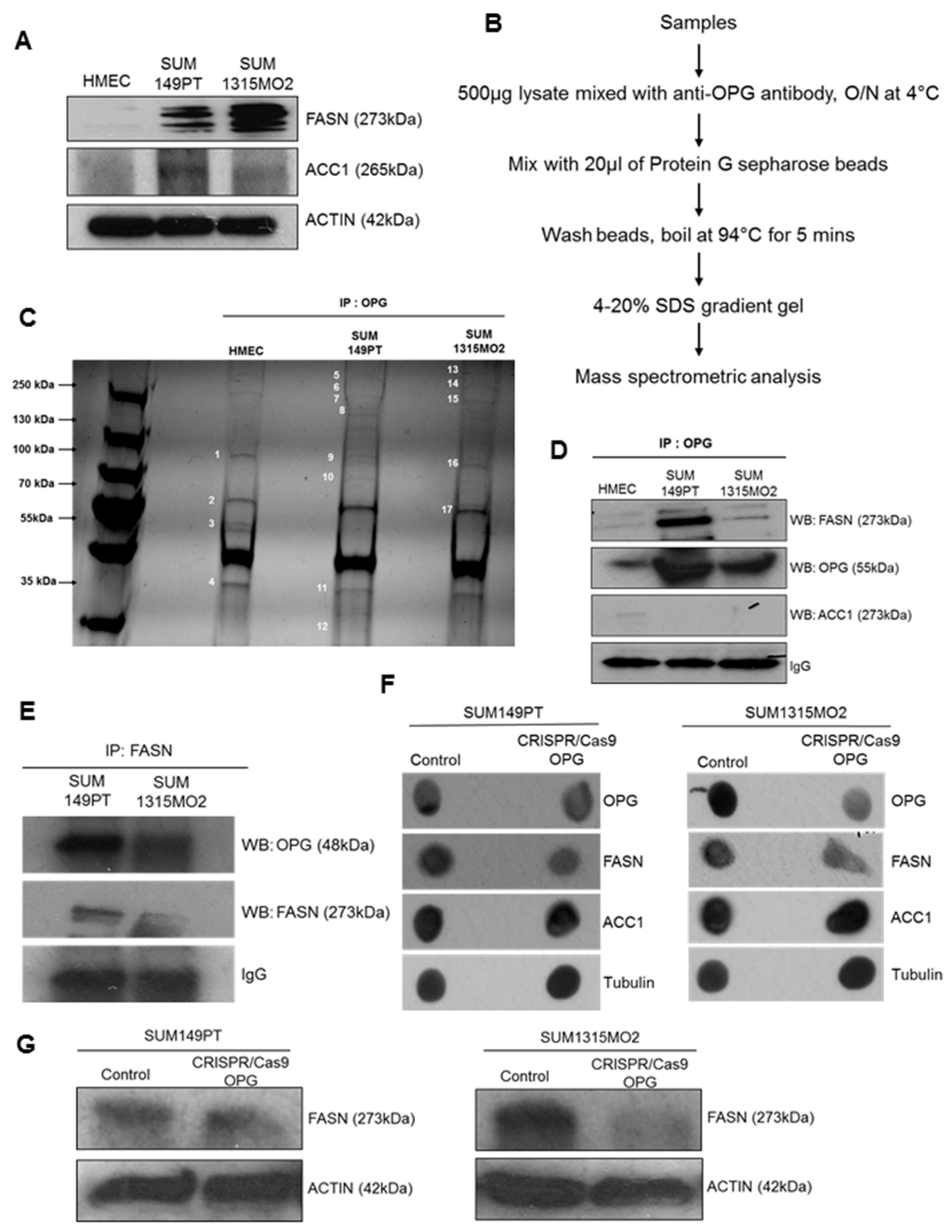
Mass spectrometric analysis of immunoprecipitates of anti-OPG with lysates from HMEC, SUM149PT, and SUM1315MO2 cells **A.** RIPA lysates from HMEC, SUM149PT and SUM1315MO2 cells were western blotted for FASN and ACC. Actin was used as a loading control. A representative blot from three independent experiments is shown. **B.** Schematic for mass spectrometric analysis **C.** Lysates from HMEC, SUM149PT and SUM1315MO2 cells were immunoprecipitated with anti-OPG antibody. Immunoprecipitated proteins were separated by SDS-PAGE and gel bands, as indicated by the numbers, were analyzed by mass spectrometry. **D.** Immunoprecipitates were subjected to Western blot analysis for FASN and OPG. Blot was then stripped and probed for ACC1 as a negative control. β-actin was used as loading control. A representative blot from three independent experiments is shown. **E.** Reverse immunoprecipitates with FASN antibody were subjected to Western blot analysis for OPG. Blot was then stripped and probed for FASN as a control. A representative blot from three independent experiments is shown. **F.** RIPA lysates from SUM149PT and SUM1315MO2 control and CRIPS/Cas9 OPG knockdown cells were dot blotted for OPG and FASN. ACC1 was used as a negative control. Tubulin was used as a loading control. A representative blot from three independent experiments is shown. **G.** RIPA lysates from SUM149PT and SUM1315MO2 control and CRIPS/Cas9 OPG knockdown cells were subjected to Western blot analysis for FASN. Actin was used as a loading control. A representative blot from three independent experiments is shown.

FASN has been shown to have a correlation with lipid body formation in cancer cells in many reports [18]. To assess if the breast cancer cells express high levels of de novo fatty acid synthesis enzymes, immunoblotting for FASN and ACC1 was performed (Figure 3A). The analysis confirmed FASN and ACC1 levels were significantly higher in SUM149PT and SUM1315MO2 cells as compared to HMEC cells (Figure 3A). Strong immunofluorescence staining for FASN and ACC1 was observed in the breast cancer cells compared to the control HMEC cells [19].

### Interaction of FASN and OPG in breast cancer cells

Our previous study [3] demonstrated that SUM149PT and SUM1315MO2 cells express and secrete high levels of OPG in their microenvironment when compared to HMEC cells. Here, we identified the OPG binding proteins in control HMEC and breast cancer SUM149PT and SUM1315MO2 cells. Cell lysates prepared from different cell types were immunoprecipitated using anti-OPG antibody, followed by LC-ESI-MS mass spectrometry analysis (Figure 3B). 17 different bands were selected for analysis (Figure 3C) and proteins were identified with a confidence range of 99.1% to 68.2% (Table 1). Comparison of each selected band displayed unique bio-signature molecules that were pulled down by OPG. These proteins belong to functionally different categories such as cytoskeleton elements, trafficking proteins, cellular chaperones, glycoprotein, enzymes, proliferation and cell cycle regulators (Table 1). The most striking was the pull-down of FASN protein by OPG immunoprecipitation. It was novel to observe the physical association of OPG with FASN for the first time in breast cancer cells. FASN catalyzes the terminal step of de novo fatty acid synthesis, without affecting other important components of lipid metabolism [20–22]. To confirm the mass spectrometry hit of FASN, we performed immunoprecipitation using anti-OPG antibody with the cell lysates prepared from HMEC, SUM149PT and SUM1315MO2 and then immunoblotted for FASN and OPG (Figure 3D). Immunoblotting for ACC1 was used as negative control to confirm that OPG binds specifically to FASN (Figure 3D). Also, to confirm these findings, reverse-immunoprecipitation using anti-FASN antibody was performed. Cell lysates prepared from SUM149PT and SUM1315MO2 cells were immunoprecipitated with anti-FASN antibody and then immunoblotted for OPG and FASN (Figure 3E). Interestingly, knockdown of OPG by CRISPR/Cas9 gene editing in breast cancer cells also decreased the FASN protein levels (Figure 3F and Figure 3G). However, no effect was observed on the ACC1 levels used as negative control (Figure 3F). These results suggest that OPG is acting upstream to FASN and playing a role in FASN regulation.

**Table 1:** Proteins identified by mass spectrometric analysis of the immunoprecipitate of anti-OPG with lysates prepared from HMEC, SUM149PT and SUM1315MO2 cells

Mass spectrometry analysis of protein immunoprecipitations with anti-OPG antibody				
Functional Category	Band No	Protein Identified	Peak Score (%)	Coverage (%)
*Cytoskeleton elements*	1	Alpha-actinin 4	99.1	25.57
	4	Actin, beta	99.1	31.25
	4	POTE ankyrin domain family member F	98.8	5.21
	5	Filamin A	99	8.34
	6	Myosin-9	98.8	9.13
	9	Alpha actinin 4	99	11.99
	9	Alpha actinin 2	98.3	5.7
	9	Alpha actinin 3	97.1	3.88
	10	Keratin 10	98.5	6.89
	11	Actin, beta	99.1	20.65
	12	Annexin A2	98.8	13.57
	13	Filamin A	99	5.23
	14	Myosin-9	99	8.78
	16	Alpha actinin 4	99.1	18.67
*Trafficking proteins*	7	Clathrin heavy chain 1	97.7	3.04
*Cellular chaperones*	2	Heat shock 70kDa protein 9 (mortalin)	99.1	30.34
	2	Bip	98.9	15.02
	3	Heat shock 60kDa protein 1 (chaperonin)	62.3	6.25
	9	Heat shock protein gp96 precursor	97.4	7.16
	10	HSP90AB1 protein	99.1	15.85
	17	Stress-70 protein, mitochondrial precursor	99.1	33.73
	17	Heat shock 70kDa protein 9 (mortalin)	99.1	32.11
	17	BiP protein	98.9	22.54
*Glycoprotein*	3	Anti-colorectal carcinoma heavy chain	98.7	8.52
	4	Anti-colorectal carcinoma heavy chain	98.8	15.28
	5,6	Fibrinogen betaB	61	10.17
	5	Fibrinopeptide B	61	85.71
	7	Anti-colorectal carcinoma heavy chain	65.6	4.37
	12	Anti-colorectal carcinoma heavy chain	99	12.01
	14	Anti-colorectal carcinoma heavy chain	98.7	8.52
*Enzyme*	6	Fatty acid synthase^*^	97.7	3.55
	6	FASN variant protein^*^	97.7	3.49
	6	Encodes region of fatty acid synthase activity; FAS^*^	97.7	3.08
	6	FASN protein^*^	67.4	7.93
	7	Glutaminyl-tRNA synthetase	98.9	8.61
	7	Ras GTPase-activating-like protein IQGAP1	98.8	9.05
	8	Isoleucine-tRNA synthetase	98.9	7.68
	12	Glyceraldehyde-3-phosphate dehydrogenase	98.4	21.79
	14	Glutamyl-prolyl-tRNA synthetase	98.7	4.63
	15	Valosin-containing protein	86.3	8.47
*Proliferation/Cell cycle*	8	Proliferation-inducing protein 44	96.2	6.19
	8	Nuclear DNA helicase II	85.7	3.15
	8	ATP-dependent RNA helicase A	85.7	3.15
	9	Nucleolin^#^	99.1	18.95
	9	Poly (ADP-ribose) polymerase family	81.6	2.88
	12	60S ribosomal protein P0	98.4	17.67
	12	NPM1 protein	68.7	6.14
	14	Ribosome-binding protein 1	98.2	3.28
	16	Nucleolin^#^	99.1	12.59
	16	NCL protein^#^	99	10.58

PEAKS score is a measure of identification confidence.

Comments:

* indicates the hit is only specific for SUM149PT cells only

# indicates the hit is specific for both SUM149PT and SUM1315MO2 cells only

### COX-2/PGE2 inflammatory pathway is upregulated in breast cancer

**Figure 4:**
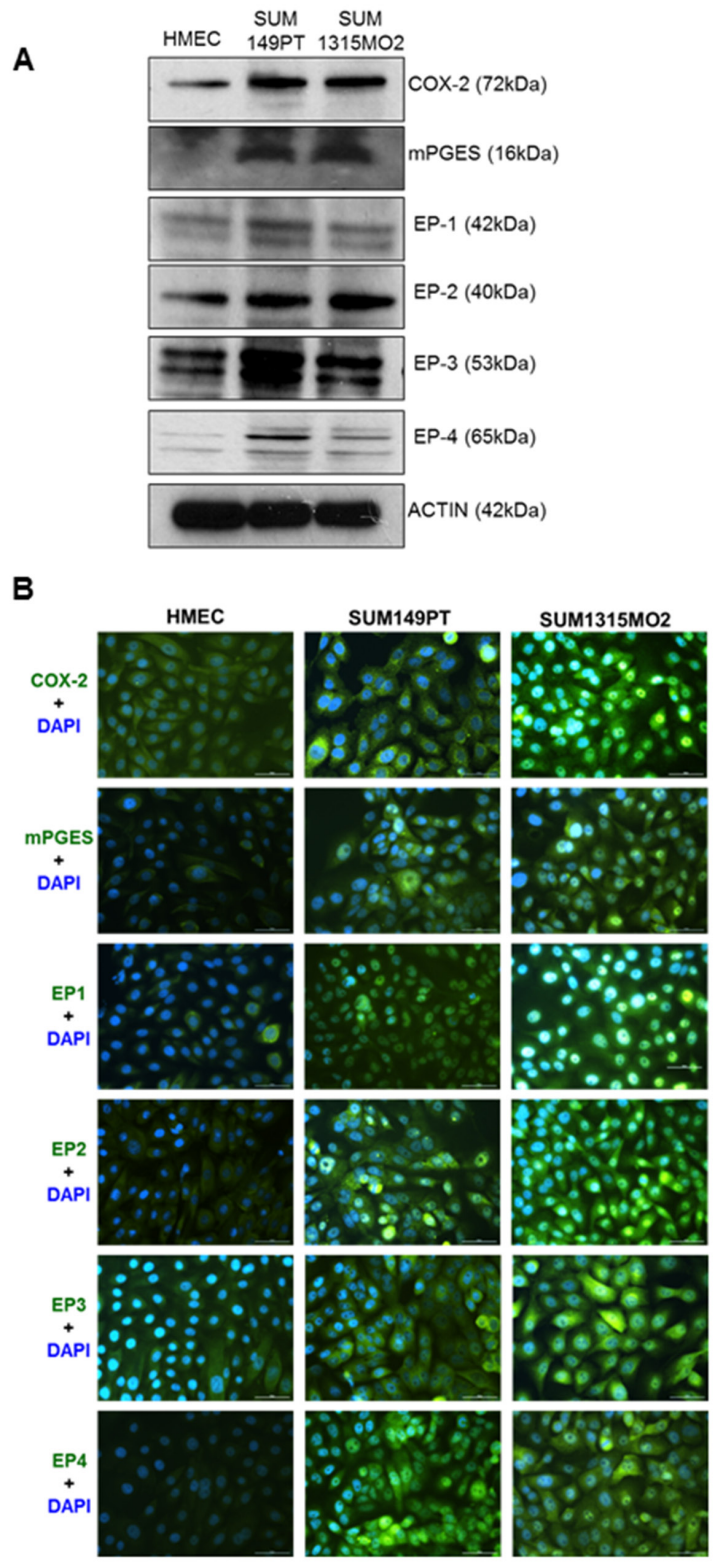
Breast cancer cells express active COX-2/PGE2 pathway **A.** RIPA lysates from HMEC, SUM149PT and SUM1315MO2 cells were Western blotted for G-protein coupled EP receptors; EP1-4, COX-2 and mPGES-1. **B.** Representative immunofluorescence images depicting distinct staining of COX-2, mPGES-1, and the EP receptors in SUM149PT and SUM1315MO2 in comparison to HMEC cells.

The elevation of FASN expression in cancer is consistent with evidences that metabolism of arachidonic acid (AA) by the COX pathway is upregulated, which plays important roles in inflammation and cancer progression [8]. Also, downregulation of FASN contributes to the chemopreventive effect of the COX-2 inhibitor celecoxib [23, 24]. Western blot analysis showed that compared to control HMEC cells, expression of the G-protein coupled EP receptors, EP1-4, COX-2 and microsomal prostaglandin E synthase-1 (mPGES-1) were significantly upregulated in the breast cancer cells SUM149PT and SUM1315MO2 (Figure 4A). mPGES-1 is the terminal synthase responsible for the synthesis of the pro-tumorigenic PGE2. The EP1 and EP4 receptors are abundantly expressed compared to EP2 and EP3 in these aggressive breast cancer cells (Figure 4A). Distinct perinuclear COX-2 staining, as well as mPGES-1, was observed in a majority of the SUM149PT and SUM1315MO2 cells in comparison to HMEC by immunofluorescence microscopy (Figure 4B). Immunofluorescence microscopy also confirmed the upregulation of all four EP receptors, EP1-4 in the breast cancer cells (Figure 4B). Additionally our recent study [19] also showed increased secretion of PGE2, a pro-inflammatory metabolite of the COX-2 pathway in the breast cancer cell microenvironment.

### FASN and COX-2 co-exist with the lipid bodies in breast cancer cells

**Figure 5:**
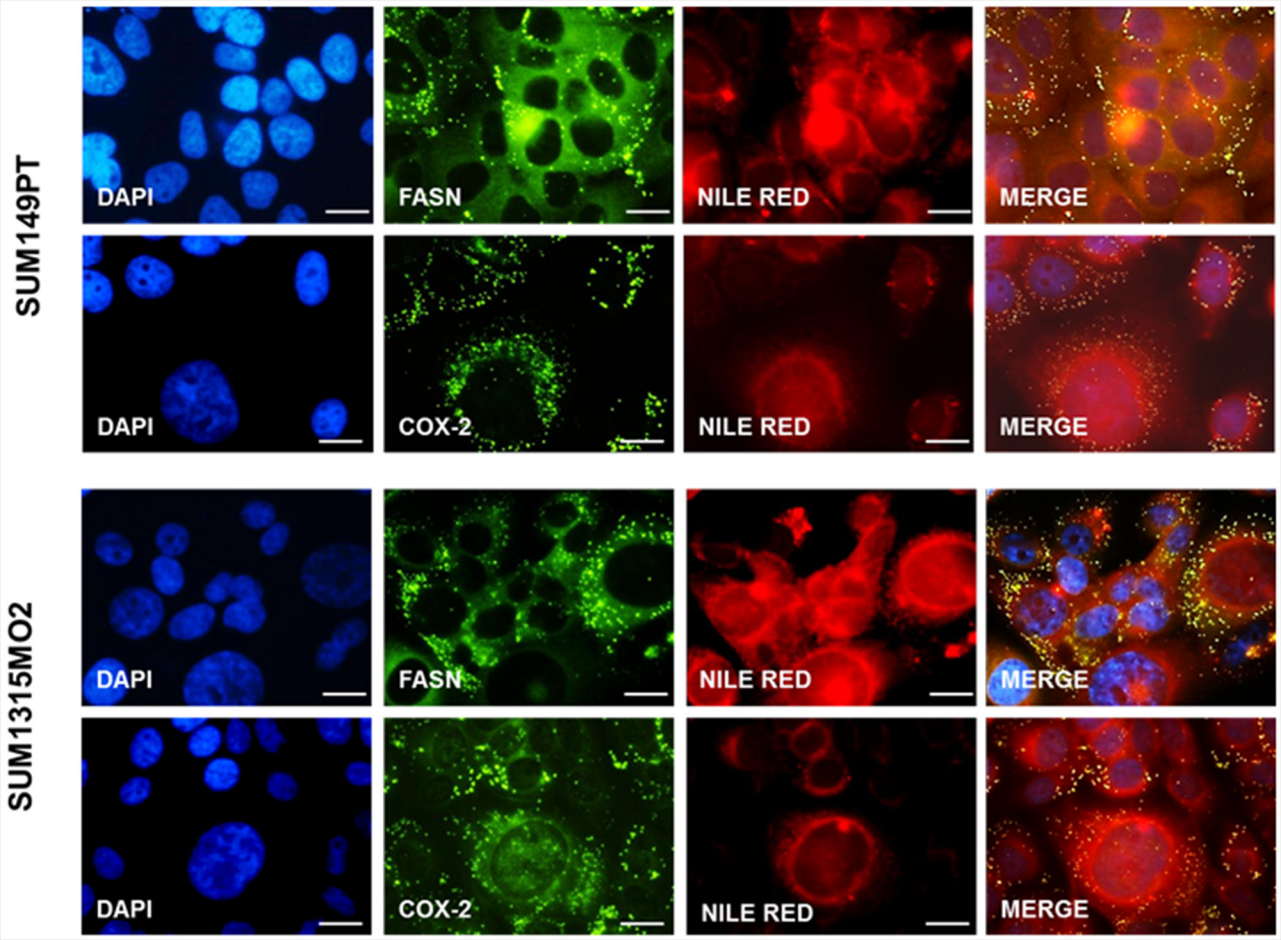
FASN and COX-2 co-exist in lipid bodies in breast cancer cells Immunofluorescence staining of FASN and COX-2 in breast cancer cells as described in the materials and methods section. Lipid bodies and nuclei were counterstained with Nile red and DAPI (blue) respectively. Magnification for the panels is 20X. The panels shown are representative images of three independent experiments. Bar represents 20μm.

Arachidonic acid metabolism by COX-2 provides one potential mechanism for explaining pro-tumorigenic effects downstream of FASN [8, 25]. Since we observed abundant lipid bodies in breast cancer cells, as well as the overexpression of COX-2 and FASN, we wanted to decipher if FASN and COX-2 co-exist with the lipid bodies in breast cancer cells. An immunofluorescence assay revealed the compartmentalization of FASN and COX-2 to the lipid bodies in the SUM149PT and SUM1315MO2 cells (Figure 5).

### OPG, FASN and PGE2 expression is significantly elevated in patient breast cancer tissue

**Figure 6:**
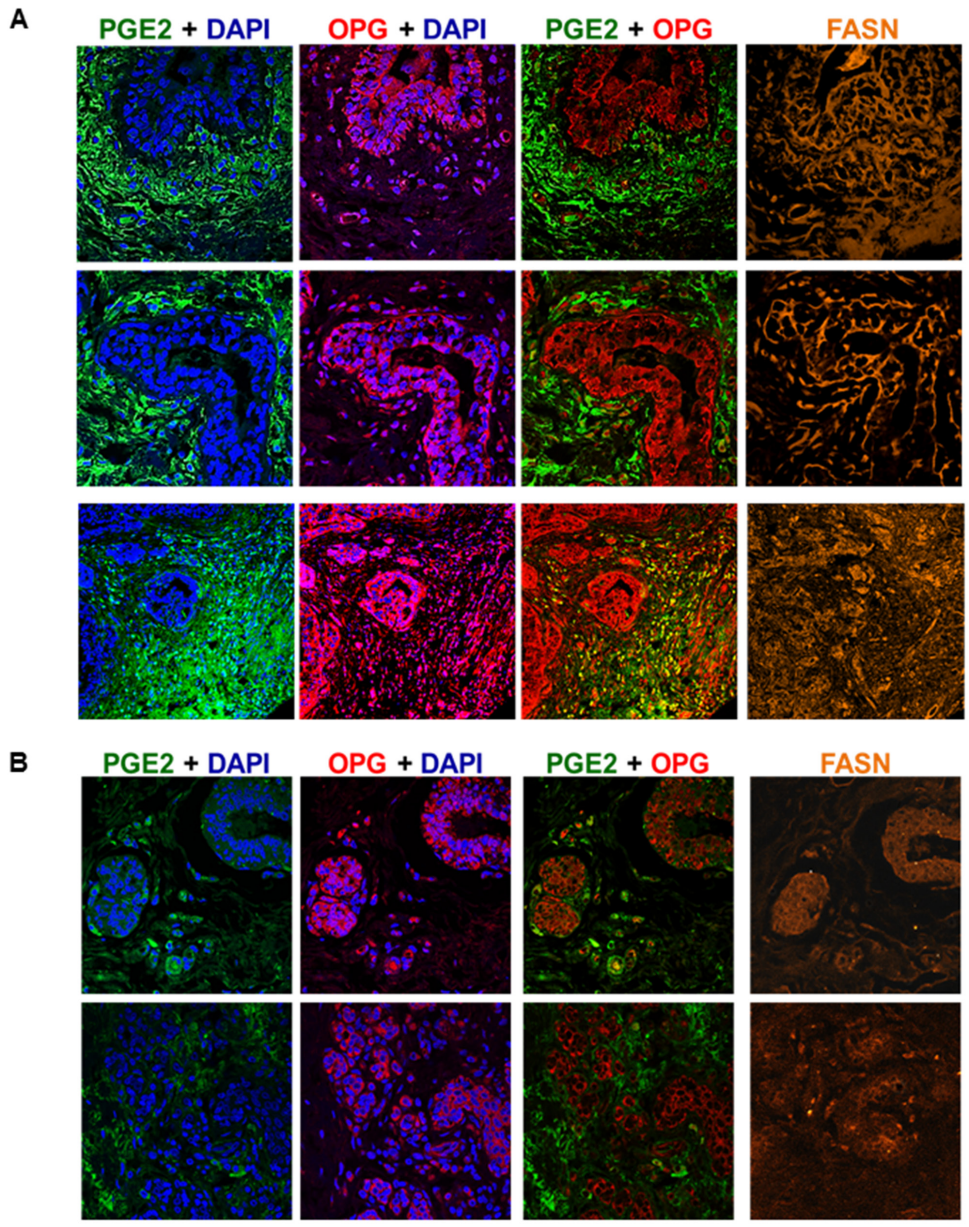
OPG, FASN and PGE2 expression is significantly elevated in patient invasive breast cancer tissue **A.** Breast cancer tissue samples were analyzed by immunofluorescence and confocal microscopy staining for OPG (red), FASN (orange) and PGE2 (green). Nuclei were counterstained with DAPI. Magnification is 60X. **B.** Normal control breast tissue samples were analyzed by immunofluorescence and confocal microscopy staining for OPG (red), FASN (orange) and PGE2 (green). Nuclei were counterstained with DAPI. Magnification is 60X.

To extend our previous *in vitro* observations, we analyzed breast tissue sections from invasive breast cancer patients for the presence of OPG, FASN and PGE2 by immunofluorescence staining (Figure 6A). Abundant OPG, FASN and PGE2 expression were detected in breast cancer tissue sections (Figure 6A). There was consistent elevated expression of FASN throughout the breast cancer tissue samples (Figure 6A). However, OPG and PGE2 were expressed in a spatial-temporal mutually exclusive manner (Figure 6A). However, little significant staining of OPG, FASN and PGE2 was observed in control breast tissue sections (Figure 6B). Collectively these results for the first time show the co-existence of OPG, PGE2 and FASN in breast cancer tissues when compared to the control breast tissues sections.

**Figure 7:**
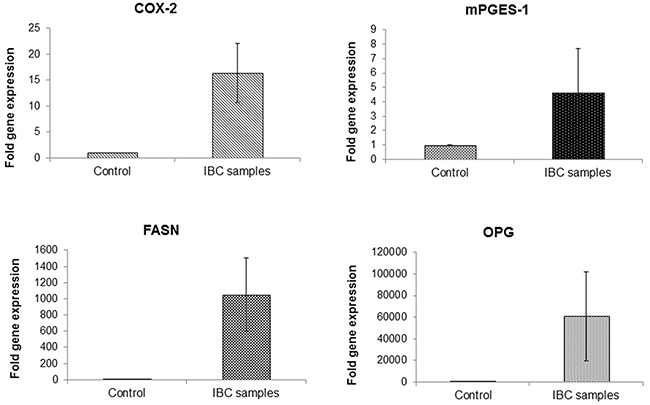
Upregulation of COX-2, mPGES-1, FASN and OPG expression in patient inflammatory breast cancer tissue cDNAs isolated from patient inflammatory breast cancer tissue and normal healthy mammary tissue samples were subjected to quantitative real time PCR analysis for the gene expression of COX-2, mPGES-1, FASN and OPG using their specific primers. Each point represents the average +/− SD from three independent experiments

We also performed RT-PCR for profiling the gene expression of OPG, FASN, COX-2 and mPGES-1 in inflammatory breast cancer patient samples. Almost ~ 15 and ~6 fold higher expression of COX-2 and mPGES-1 was observed in IBC samples when compared to control normal human mammary tissue samples(Figure 7). Remarkably very high gene expression of OPG and FASN was observed in IBC samples in comparison to normal healthy samples (Figure 7).

### Effect of OPG on COX-2 promoter transcription regulation via NF-κB regulatory element

**Figure 8:**
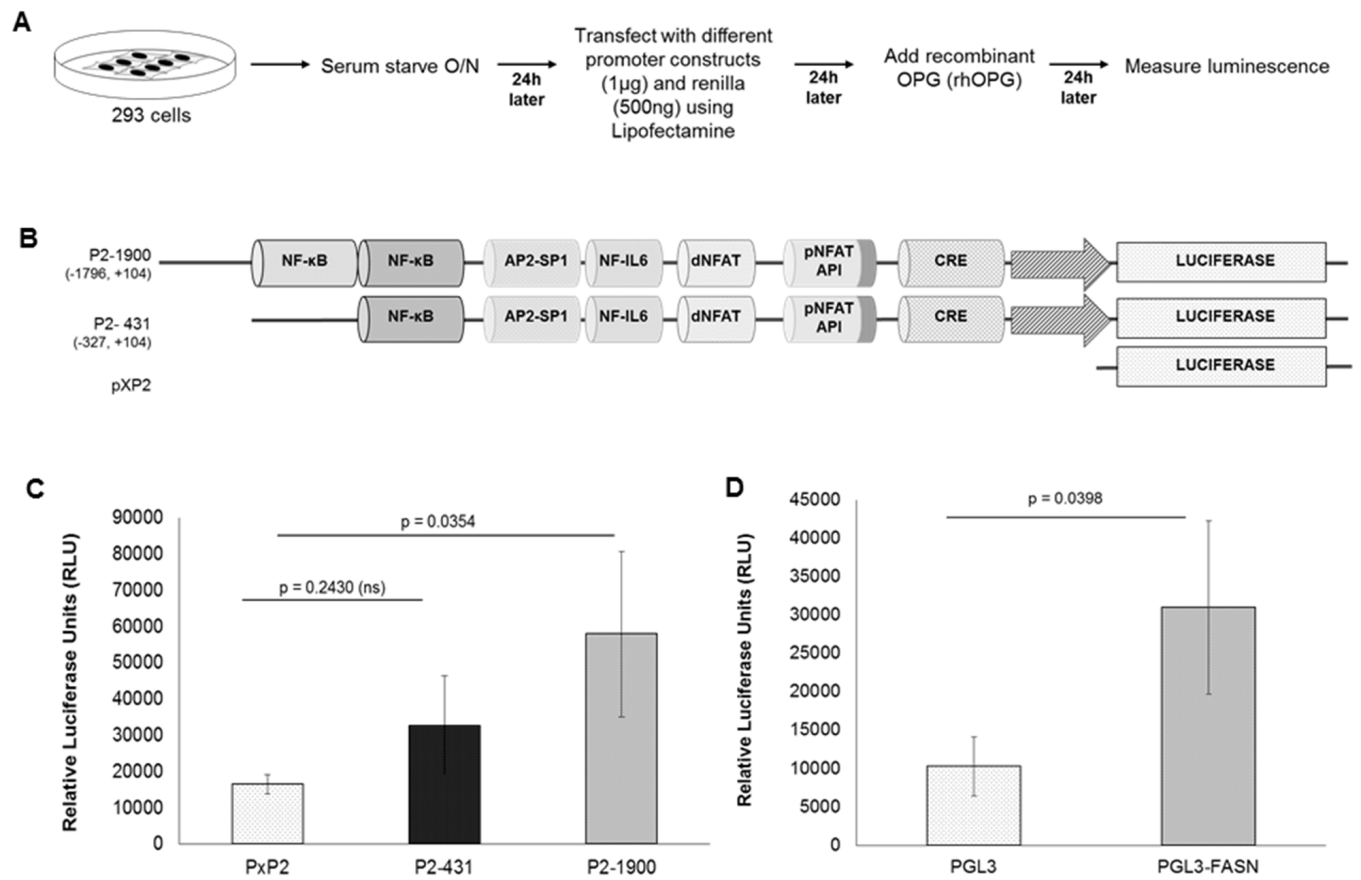
Role of cis-acting factors in transcriptional regulation of the COX-2 and FASN promoter by recombinant OPG **A.** Schematic of transfection and luciferase assay. **B.** Schematic of COX-2 promoter constructs. The extent of 5′ deletions is shown with the numbers indicating their lengths relative to the transcription start site. Transcription factor binding sites are represented on the promoter, while the promoter regions relative to the transcription initiation start site are shown in parentheses. **C.** 293 cells were transfected with control PXP2 or various COX-2 promoter constructs P2-1900-luc and P2-431-luc. 24 h after transfection, 500 pg/ml rhOPG was added to the transfected cells for 24 h followed by luciferase assay. **D.** 293 cells were transfected with control pGL3 or full-length pGL3-FASN promoter constructs. At 24 h after transfection, 500 pg/ml rhOPG was added to the transfected cells for 24 h. These cells were lysed, and luciferase assay was performed. Data in panels C, and D represent the mean number of RLU after normalization with the cotransfected Renilla luciferase activity. Each reaction was done in triplicate and each point represents the average +/− s.d. of three independent experiments.

It is well known that COX-2 promoter activity is regulated in other systems by several transcription factors, including NF-kB, nuclear factor IL-6, AP-1, CRE, and NFAT [26]. OPG is closely related to NF-kB [27]. To understand the role of NF-κB and its involvement in COX-2 upregulation by OPG we studied the role of OPG on the different constructs of the human COX-2 promoter (Figure 8C). Details of the different COX-2 promoter luciferase constructs have been described previously [28, 29] and their maps are given in Figure 8B. In the presence of OPG, the activity of the COX-2 full-length promoter luciferase construct (P2-1900) increased by 3.5 fold when compared to that of the pXP2 control construct. Interestingly, activation of P2-431 (construct with one NF-kB site deleted) was reduced by 1.78 fold as compared to P2-1900, which has both NF-kB sites (Figure 8C). These results suggest that OPG induced NF-kB plays an important role in regulating the transcriptional activation of COX-2. The possibility of regulation via other transcription factors could not be ruled out as the full-length promoter has multiple transcription factor binding sites such as proximal NFAT, distal NFAT, AP-1, and IL-6 [26].

### Effect of OPG on the FASN promoter transcriptional regulation

To investigate the role of OPG on FASN gene transcription regulation, FASN full-length promoter (pGL3-FASN) and control pGL3 construct were used to transfect 293 cells overnight. OPG was added to the transfected cells for 24h. The 3-fold increase in luminescence intensity (Figure 8D) of pGL3-FASN when compared to that of pGL3 suggests the involvement of OPG in the upregulation of FASN gene transcription.

### Celecoxib and C75 induces cell apoptosis via caspase 9/3 dependent pathway

**Figure 9:**
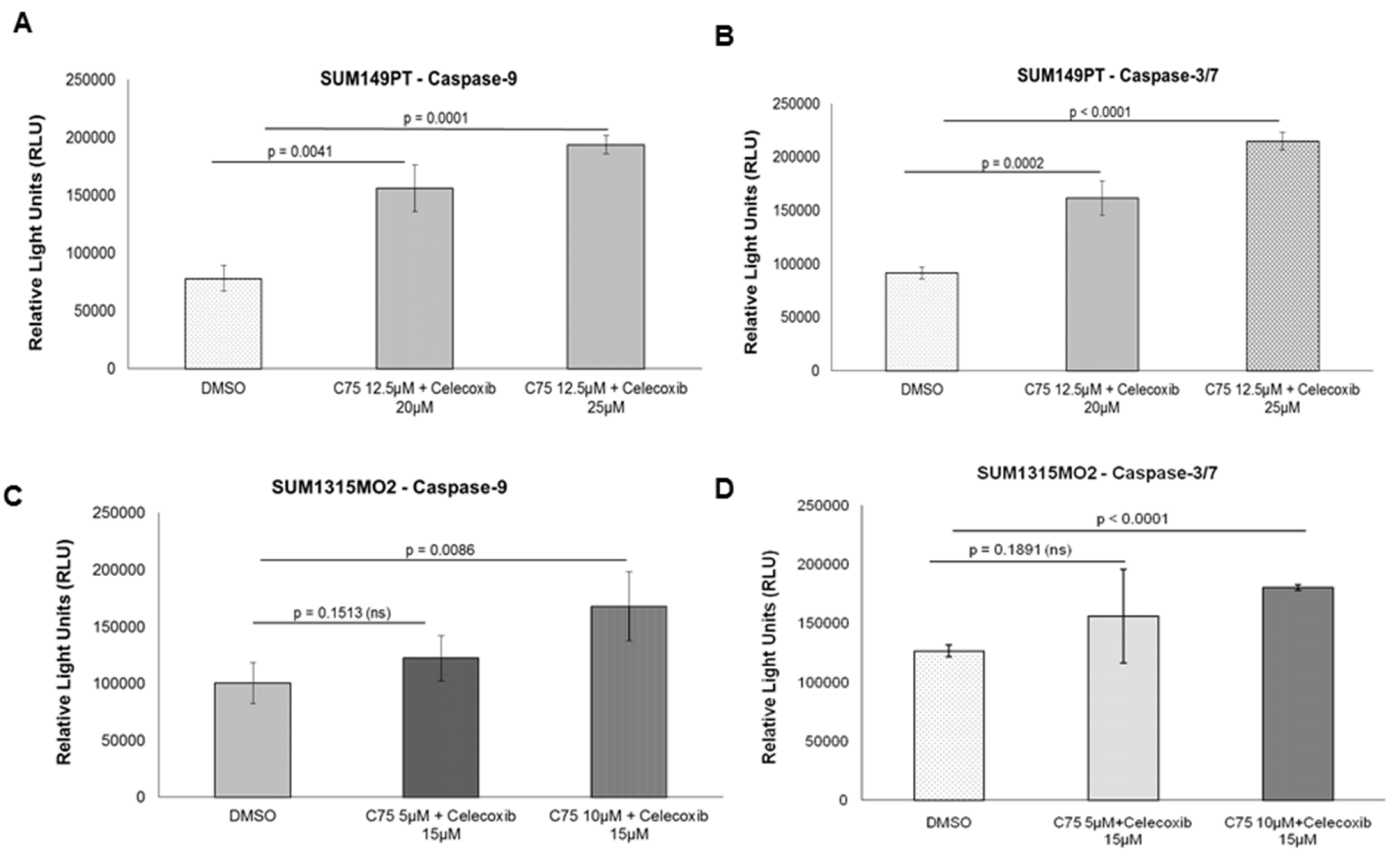
Apoptosis in breast cancer cells by C75 and celecoxib via caspase pathway **A.** and **B.** SUM149PT and **C.** and **D.** SUM1315MO2 cells were plated in a 48-well plate. Upon confluency they were treated with respective C75 and celecoxib drug concentrations for 24h. After 24h, the cells were lysed in respective buffer and luminescence was measured.

Since SUM149PT and SUM1315MO2 cells have upregulated FASN and COX-2 pathways, we screened the effects of FASN inhibitor C75 and COX-2 inhibitor celecoxib or both in combination on the breast cancer cell lines using caspase 9 and caspase 3/7 assays. At 1d post-treatment 12.5μM C75 had induced significant apoptosis in SUM149PT cells (Figure 9A and 9B) when compared to DMSO. A similar trend was observed by using 25μM celecoxib for treating SUM149PT cells (Figure 9A and 9B). Combining 12.5μM C75 and 25μM celecoxib for treating SUM149PT cells had a profound superior effect on apoptosis of the cells in comparison to DMSO (Figure 9A and 9B and Supplementary Figure S1). For SUM1315MO2 cells, 10μM C75 and 15μM celecoxib had induced significant apoptosis (Figure 9C and 9D) when compared to DMSO. A more robust effect was observed on combining 10μM C75 and 15μM celecoxib for treating SUM1315MO2 cells (Figure 9C and 9D and Supplementary Figure S1). Overall, based on the drug treatment comparison analysis, we chose 12.5μM C75 and 25μM celecoxib as the drug concentration for treating SUM149PT and 10μM C75 and 15μM celecoxib as the drug concentration for treating SUM1315MO2 throughout our study.

### Combinatorial C75 and celecoxib treatment lowers lipid body count in breast cancer cells

**Figure 10:**
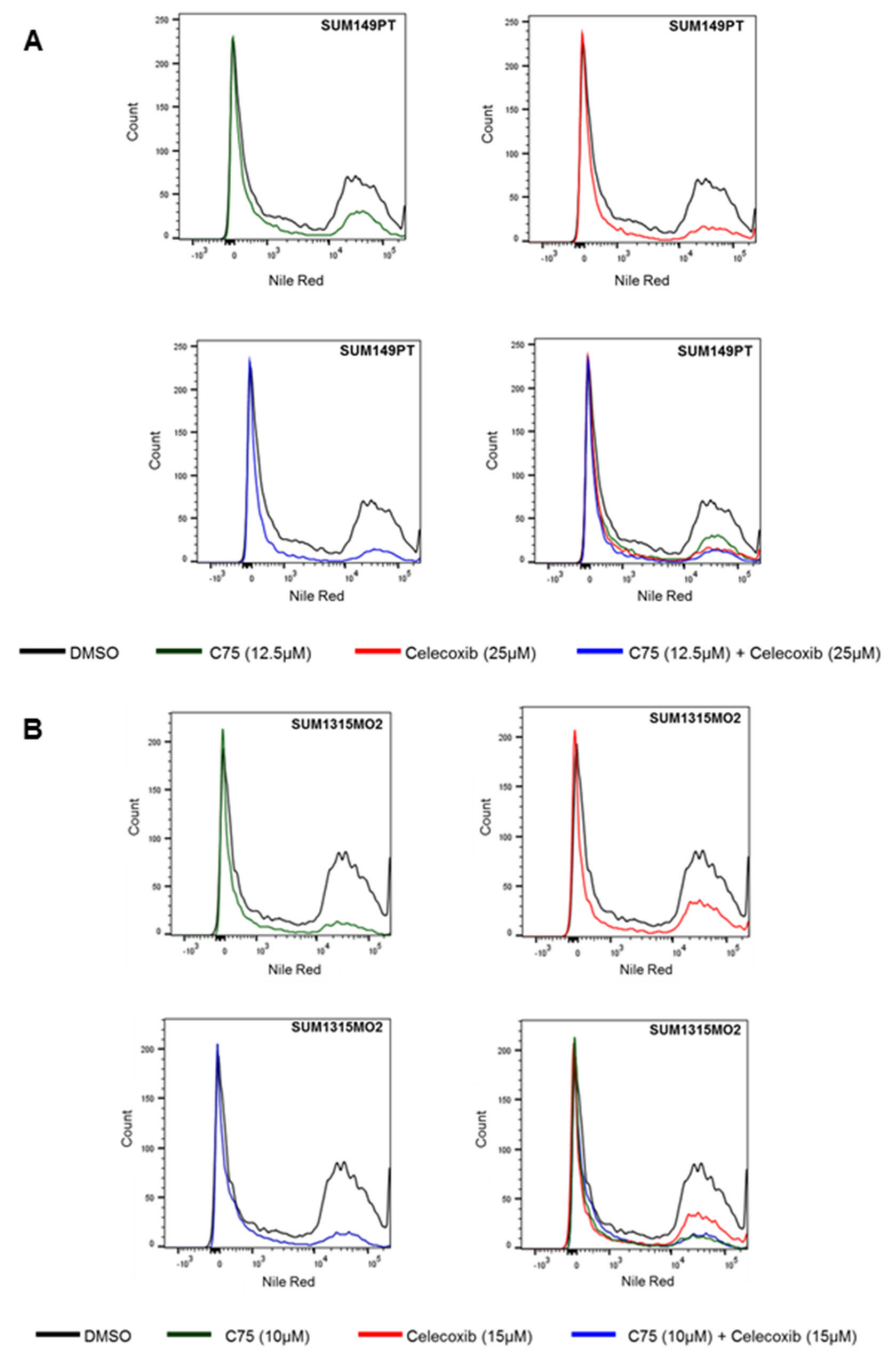
Combinatorial C75 and celecoxib treatment lowers lipid body count in breast cancer cells Nile red staining of DMSO, C75, celecoxib and C75 + celecoxib treated **A.** SUM149PT and **B.** SUM1315MO2 breast cancer cells was done and flow cytometry analysis was performed. Data were analyzed using FlowJo software. The panels shown are representative images of three independent experiments.

Lipid body formation is important during breast cancer progression and inflammation [30]. Hence, we hypothesized that if we block the inflammatory pathways, we may potentially limit lipogenesis during breast carcinogenesis and its progression. We treated SUM149PT and SUM1315MO2 cells with either C75, celecoxib individually or in combination. Following drug treatment, we quantitated lipid body formation by staining the cells with a lipophilic lipid-binding dye called Nile red and performed flow cytometry. In SUM149PT, as compared to DMSO control, when cells were treated with either C75 or celecoxib there was a reduction in the amount of lipids formed per cell (Figure 10A). In the presence of C75 or celecoxib treatment alone, 27.9% and 25.9% of cells respectively were positive for lipid bodies in comparison to 41.7% of DMSO treated cells. The SUM149PT breast cancer cells seems to be primarily dependent on the COX-2 pathway as combinatorial treatment with celecoxib reduced the lipid body positive cells to 25.9% in comparison to 27.9% lipid body positive cells when treated with C75. Interestingly, in invasive breast cancer SUM1315MO2 cells, C75 reduced the lipid body positive cells to 23.4% in comparison to 37.2% lipid body positive cells when treated with celecoxib (Figure 10B). In contrast, in SUM1315MO2, treatment with C75 and celecoxib reduced the lipid body positive cells to 23.4% and 37.2% in comparison to 53% of DMSO treated cells (Figure 10B). This highlights the dependence of SUM1315MO2 cells on the fatty acid synthesis pathway. Overall, it is evident that when both drugs were used for treatment, a greater reduction could be observed in the amount of lipids formed per cell. Thus, it could be inferred that the drugs shows superior activity in breast cancer treatment by targeting various inflammatory pathways and hence this can be translated into a long-term treatment for patients.

### Celecoxib and C75 downregulate cell survival kinases in breast cancer cells

**Figure 11:**
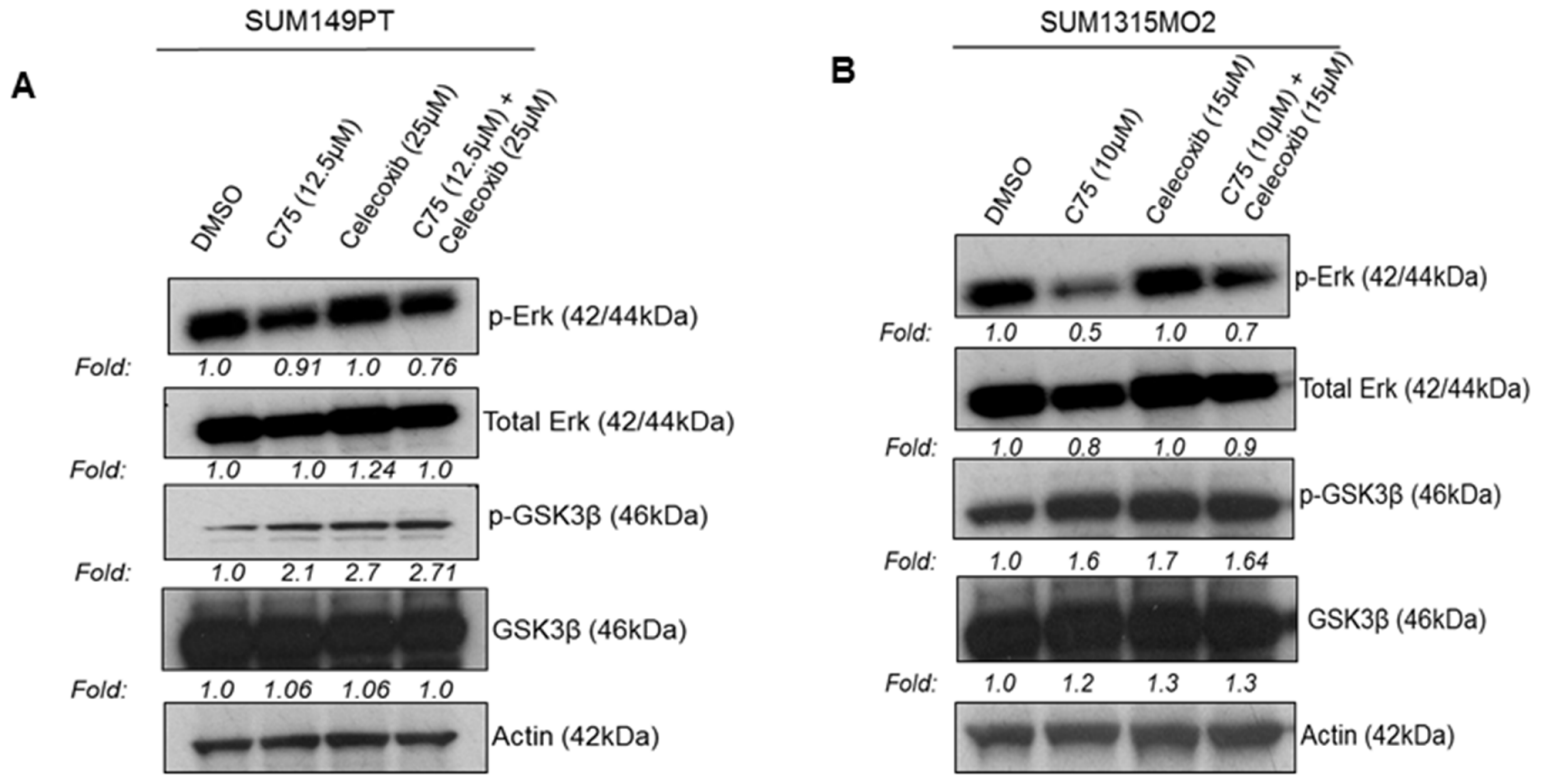
Effect of C75 and celecoxib on the survival kinases in breast cancer cells **A.** SUM149PT and **B.** SUM1315MO2 breast cancer cells were treated with DMSO solvent, C75 or celecoxib, alone or C75 and celecoxib combined. Cell lysates were Western blotted using p-Erk and p-GSK3β antibodies. These blots were stripped and blotted with total antibodies against Erk and GSK3β. Actin was used as the loading control.

We next examined the effect of blocking FASN and COX-2 on major cell survival proteins such as p-Erk and p-GSK3β. Total cell lysates of SUM149PT and SUM1315MO2 cells treated with either C75, celecoxib or C75 and celecoxib together were collected at 24 h and used to measure p-Erk and p-GSK3β activation levels. Erk and GSK3β phosphorylation levels were decreased in the breast cancer cells when treated with the drugs (Figures 11A and 11B). In SUM149PT, combinatorial treatment was highly effective in decreasing levels of p-Erk and p-GSK3β when compared to that of just C75 or celecoxib alone. Contrastingly, in SUM1315MO2, C75 was more effective in decreasing levels of p-Erk and p-GSK3β when compared to that of celecoxib. Hence, we can say that in SUM1315MO2, the effect that we see with combinatorial drug treatment is primarily attributed to C75.

## DISCUSSION

We recently reported that the breast cancer microenvironment is rich in OPG, which can influence healthy HMEC cells and drive them towards tumorigenesis [3]. Our study demonstrated that OPG induces proliferation, angiogenesis, aneuploidy and survival through the manipulation of various survival and aneuploidy related kinases in normal healthy HMEC cells [3]. Here, we demonstrated several interesting findings in the context of paracrine action of the OPG rich breast cancer microenvironment that drives carcinogenesis via inducing and sustaining inflammatory COX-2 and lipogenic FASN in an invasive breast cancer setting. Our study revealed the superior anti-proliferative role of COX-2 inhibitor celecoxib and FASN blocker C75 in aggressive breast cancer cells.

### Importance of lipid metabolism and lipid bodies in breast cancer

As the malignant transformation of cells requires adaptations across multiple metabolic processes to meet the energy required for the increased rate of proliferation, deregulation of metabolism has been a hallmark event [31]. Cancer cells feature increased oncogenic de novo lipogenesis [32]. Increased lipid accumulation in the form of lipid bodies/lipid droplets and upregulation of various lipogenic enzymes, as well as of enzymes that function to oxidize fatty acids as an energy source are common phenomenon of cancers, including prostate [33], liver [26], and colorectal cancer [30]. The lipid profiling of breast cancer cells has been reported to discriminate metastatic and non-metastatic cancer phenotypes [34]. Accordingly, we observed a remarkable number of lipid bodies in SUM149PT and SUM1315MO2 breast cancer cell lines.

Besides an increase in lipid bodies there are other organelle changes in breast cancer cells, which can contribute to the metabolic dysregulation. EM images clearly depicted that breast cancer cells have a high concentration of mitochondria located within close proximity of the lipid bodies. This supports the observed physiological roles of mitochondria in coordinating lipid metabolism and in controlling reactive oxygen species (ROS), ATP and calcium levels, several factors that influence the aggressiveness of the disease [35].

Fatty acid synthesis, a process of producing de novo fatty acids from carbohydrate and amino acid derived carbon sources, is controlled by the FASN enzyme [6, 36]. Overexpression of FASN allows for de novo synthesis of essential lipids for the formation of cell membranes and for the production of extra energy via beta-oxidation and lipid modification of proteins [37]. It is a multifunctional polypeptide enzyme that produces saturated fatty acids, uses one acetyl-CoA and sequentially adds seven malonyl-CoA molecules to produce the 16-carbon saturated palmitic acid and thus plays a crucial role in maintaining lipid homeostasis [6, 36]. Interestingly, overexpression of FASN has also been strongly associated with many biologically aggressive cancer types and is under extensive study as a potential cancer drug target [6, 36, 38, 39, 40]. FASN is minimally expressed in most normal human tissues [41, 42] but it is an essential component of mammary gland physiology during lactation [6, 36]. Upregulation of FASN gene expression is an early event in cancer development that is more pronounced in advanced tumors [41, 42]. FASN makes an attractive therapeutic target as it appears to be upstream of multiple neoplastic transformations and metastasis as well as angiogenic pathways manipulating tumor vascularity and cell proliferation [43–49]. FASN also serves as a potential diagnostic and prognostic biomarker as it is secreted in the blood of patients with breast, prostate, colon and ovarian cancers compared with normal healthy subjects [43]. Excitingly, we observed high expression of FASN in breast cancer cell lines in contrast to human mammary epithelial cells.

It has been reported that triglyceride metabolism in bone tissue is associated with osteoblast and osteoclast differentiation controlling genes like lipoprotein lipase (LPL), hormone sensitive lipase (HSL), FASN, adiponectin, RUNX2, RANK, RANKL and OPG [50]. Mass spectrometry analysis identified FASN as one of the binding partners for OPG in breast cancer cells. Thus by interacting with a potential integrative metabolic mediator FASN, OPG can indirectly modulate the metabolism and modification of proteins in an aggressively dividing cancer cell. Two unique characteristics of FASN, such as its tissue distribution and its enzymatic activity, make it suitable for an antitumor target.

### Upregulation of the COX-2/PGE2 inflammatory pathway in inflammatory breast cancer and its connection with OPG and FASN

In inflammatory cells, lipid bodies have been investigated for important roles in regulating arachidonic acid (AA) metabolism. AA, an essential polyunsaturated fatty acid with signaling functions and the precursor of prostaglandins and leukotrienes, is proposed to be stored in its esterified form in lipid bodies [51–54]. In our previous studies, we observed a significantly high level of prostaglandin E2 in the conditioned media of IBC cell lines, which was accompanied by an increased expression of COX-2 in these cell lines [3]. Our study revealed that increased FASN expression was accompanied by an active inflammatory COX-2 pathway in the breast cancer cells. This is in accordance with previous research findings which highlight that extranuclear lipid bodies are the sites where different enzymes involved in prostaglandin synthesis including cPLA2 and COX-2 are located [51, 55, 56]. Immunofluorescence staining revealed that in addition to perinuclear COX-2 and prostaglandin E2 (PGE2) synthase staining, there was tremendous punctate cytoplasmic staining that was concordant with Nile red staining of lipid bodies.

Levels of COX-2 are tightly controlled in most tissues, and its gene regulation is exclusively dependent on gene transcription and post-transcriptional events [10]. High levels of FASN, OPG and COX-2/PGE2 expression in human breast cancer tissue sections and breast cancer cells suggested that there must be a loop among these tumorigenic factors.

### Paracrine role of osteoprotegerin (OPG) in transcriptional regulation of COX-2 and FASN or vice versa

Since breast cancer cells express high levels of OPG, FASN, and COX-2, we asked if the OPG rich microenvironment drives the transcription of COX-2 and FASN. It is reported that PGE2 at a low dose switches osteoblast biology in favor of bone apposition by inducing significantly higher OPG gene expression [57, 58]. Interestingly, there are reports suggesting that the osteoclastogenic effect of PGE2 is markedly decreased when the osteoblasts are derived from cells lacking PGE2 receptors EP2 and EP4 [59].

COX-2, discovered in 1991 as a primary response gene, is regulated by both transcriptional and post-transcriptional mechanisms. Human COX-2 gene transcription is primarily regulated by the cAMP response element (CRE), the CAAT/enhancer binding protein (C/EBP-NF-IL6), nuclear factor of activated T cells (NFAT), NFkB sites, and the E-box [60, 61]. We identified OPG induced NF-KB as one of the important transcription factors involved in the transcriptional upregulation of COX-2. OPG present in the breast cancer microenvironment has the significant potential to stimulate the transcription of FASN. FASN can be regulated by other lipid metabolism associated genes such as SREBP-2, SREBP1c, Acyl-CoA, CPT1a and HMG-CoA synthase [19]. There is a very high probability that downregulation of FASN expression by OPG CRISPR/Cas9 knockdown (as observed in Figure 3) might be due to an effect on these lipid metabolism genes which in turn would affect the lipid content in the breast cancer cells. Since elevated FASN level as well as OPG level have been identified in the blood of patients with various cancers, it is possible that the physical association of OPG and FASN,as observed by pulldown assay in Figure 3, is aiding in each other's secretion.

### Significance of combinatorial drug targeting in breast cancer

**Figure 12:**
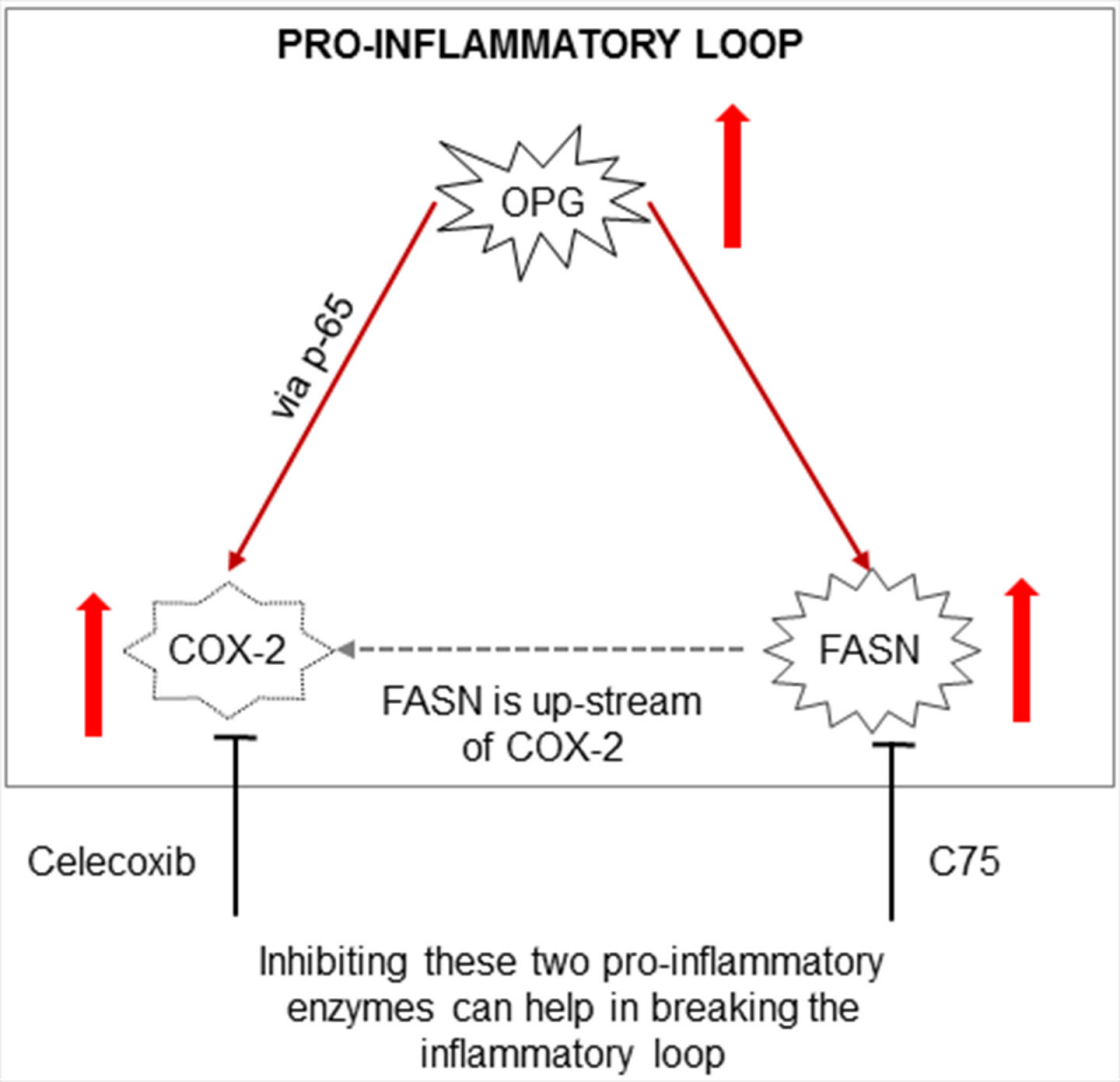
Schematic model depicting the potential pro-inflammatory loop in breast cancer cells OPG induces the transcriptional regulation of FASN and COX-2 expression. Blocking the downstream effects of FASN and COX-2 using C75, Celecoxib or both can help break this ongoing inflammatory loop thus inhibiting downstream cellular effects such as inducing cell apoptosis and decreasing cell proliferation and survival.

A novel positive feedback loop involving FASN/p-ERK1/2/5-LOX/LTB4/FASN sustains high growth of breast cancer cells (MCF-7 and LM-MCF-7; metastatic subclone of MCF-7 breast cancer cell line) [62]. Lapatinib alters the malignant phenotype of osteosarcoma cells via downregulation of the activity of the HER2-PI3K/AKT-FASN axis *in vitro* [63] suggesting that targeting signaling and the FASN axis could be detrimental for cancer cell survival. Here, we hypothesized that co-expression of FASN, COX-2, and OPG might be responsible for poor prognosis breast cancer and adds to the severity and malignancy associated with breast cancer (Figure 12). FASN expression is often upregulated in rapidly proliferating cells [64]. Inhibition of FASN expression could repress cell proliferation in various cancers [65, 66]. Thus, FASN has become an attractive target for cancer therapy in last 15 years [67]. Compelling studies have demonstrated the effect of aspirin and other non-steroidal anti-inflammatory drugs (NSAIDs) or specific COX-2 inhibitors to block PGE2 synthesis, carcinogenesis, proliferation, angiogenesis, and inflammation in cancers of the colon, esophagus, lung, bladder, breast and prostate [68, 69–71]. With this knowledge, we evaluated the effect of FASN and COX-2 inhibition on lipid body formation. Treatment with either celecoxib or C75 significantly reduced the number of lipid bodies and PGE2 production in IBC cells, and this is encouraging, as it could have added therapeutic potential in aggressive breast cancer. This study serves as the basis for future studies designed to validate the in vivo anti-tumorigenic efficacy of COXIB and FASN inhibitor combination therapy in breast cancer models (Figure 12).

## MATERIALS AND METHODS

### Cells

Primary human mammary epithelial cells (HMEC) (830-05a, Cell Applications, San Diego, CA) were cultured in HMEC medium (815-500, Cell Applications). Primary inflammatory breast cancer cells, SUM149PT (Asterand, Detroit, MI), and highly invasive breast cancer cells, SUM1315MO2 (Asterand), were grown in F-12 media (11765-054, Gibco BRL, Grand Island, NY) supplemented with 10% heat-inactivated fetal bovine serum (HyClone, Logan, UT), insulin (19278, Sigma, St. Louis, MO), HEPES (H3375; Sigma), EGF (E9644; Sigma) for SUM1315MO2 and Hydrocortisone (H4001, Sigma) for SUM149PT. All cells were tested for mycoplasma contamination by the standard Limulus assay (Limulus amebocyte lysate endochrome; Charles River Endosafe, Charleston, S.C.) method as per manufacturer's instructions. All cells were cultured in LPS-free medium.

### Inhibitors

Celecoxib (N-(5-Acetyl-2-piperidinophenyl)-N′-(2, 5-dichlorophenyl) thiourea) (Calbiochem/Life Science, Massachusetts) is a selective COX-2 inhibitor. C75 (4-Methylene-2-octyl-5-oxotetrahydrofuran-3-carboxylic acid) (Santa Cruz Biotechnology, Dallas, Texas) is a selective inhibitor for FASN.

### Reagents

Antibodies against FASN, ACC1, COX-2, Erk, p-Erk, GSK3β and p-GSK3β were from Cell Signaling. Antibodies against m-PGES, COX-1, EP-1, EP-2, EP-3, and EP-4, were from Cayman Chemicals, Ann Arbor, MI. Antibody against OPG was from Abcam while the antibody against β-actin was from Sigma. Nile Red (151744, MP Biomedicals, Solon, OH) was dissolved in ethanol for staining lipid bodies.

### Immunofluorescence Assay (IFA)

HMEC, SUM149PT, and SUM1315MO2 cells were seeded in eight-well chamber slides,(Nalge Nunc International, Naperville, IL.) fixed with 4% paraformaldehyde, permeabilized with 0.4% Triton X-100, and stained with primary antibody overnight at 4°C. Cells were washed and developed with Alexa 594 or Alexa 488-coupled secondary antibody (Molecular Probes, Eugene, OR), and nuclei were visualized using DAPI (Ex358/Em461; Molecular Probes) as counter stain. Stained cells were washed and viewed with the appropriate filters on an Olympus confocal laser-scanning microscope (Fluoview FV10i) with the Metamorph digital imaging system [72].

### Immunohistochemistry (IHC)

Sections of breast tissue from healthy subjects and cancer patients were obtained from Biochain Institute, Inc. (breast tumor tissue array Z7020007). The 16 patient breast cancer tissues array was used in an attempt to highlight a specific type of breast cancer i,e the invasive ductal carcinoma. The sample distribution was hugely varied with the tumor staging from T1N0M0 to T4N1M0 (T indicates the primary tumor, N indicates the regional lymph node metastasis and M indicates distant metastasis), the tumor grade varying from grade I-III and the age of the patients ranging from 28 – 77. IHC was performed using primary antibodies against human OPG, FASN and PGE2 using the protocols as described previously [72].

### Western blot analysis

Cell lysates were quantitated by BCA assay and equal amounts of protein (40μg/lane) were separated on SDS-PAGE, electrotransferred to 0.45-μm nitrocellulose membranes, blocked with 5% BSA, probed with the antibodies of interest, and visualized using an enhanced-chemiluminescence detection system [72].

### Luciferase reporter assays

Effect of OPG on COX-2 full length promoter (P2-1900), deletion, or mutant constructs [28, 29] was measured using a Dual-Luciferase kit according to the manufacturer's protocol (Promega, Madison, WI). 293 cells were transfected using lipofectamine. The relative COX-2 promoter activity or number of relative luciferase units (RLU) was normalized to Renilla protein levels. Similarly, we performed the analysis for the effect of OPG on the transcriptional regulation of FASN promoter (pGL3-FASN). A plasmid encoding firefly luciferase under the control of the wild-type (WT) FASN promoter was obtained from Qiang Liu (Western College of Veterinary Medicine, University of Saskatchewan, Saskatoon, SK, Canada) and has been described previously [62].

### Lipid body staining

HMEC, SUM149PT, and SUM1315MO2 cells seeded in eight-well chamber slides (Nalge Nunc International) were fixed with 4% PFA, and stained with lipophilic stain Nile red (Nile blue oxazone) and counterstained with DAPI. Nile red is an excellent vital stain for the detection of intracellular lipid bodies. Stained cells were washed and viewed with the appropriate filters on an Olympus confocal laser-scanning microscope at 450/500nm excitation and 528nm emission (Fluoview FV10i) with the Metamorph digital imaging system [72].

### Lipid body analysis by flow cytometry

To determine the intracellular lipid content of control HMEC and breast cancer cells SUM149PT and SUM 315MO2 (treated and untreated), samples were incubated with Nile red (1 μg/ml) in the dark for 5 min at room temperature. The fluorescence intensity of each sample was measured immediately by flow cytometry at an excitation wavelength of 488 nm and emission wavelength of 550 nm. The data were collected using an LSRII flow cytometer and analyzed with FlowJo software at the RFUMS flow cytometry core facility.

### Gene expression analysis by RT-PCR

Total RNA was isolated with TRIzol Reagent (Life Technologies Corporation, Grand Island, NY) from inflammatory breast cancer tissue samples (Biochain, breast tumor tissue array T22350862-2) and treated with DNase I (Life Technologies Corporation) at 37°C for 30 min. Reverse transcription was performed using a High-Capacity cDNA reverse transcription kit (Life Technologies Corporation) and converted to cDNA, relative abundance of target gene mRNA was measured by qRT-PCR using the deltadelta method (ratio, 2[DCt sample-DCt control]) as described previously [73]. Transcripts of the genes of interest were detected by real-time RT-PCR using gene- specific primers as per procedures described previously [73]. Normalization was done with respect to GAPDH mRNA levels.

### Transient transfection

For reporter gene assays, 293 cells were transfected using Lipofectamine 2000 as described before [74].

### OPG knockdown in breast cancer cell lines

A pool of three plasmids was obtained from Santa Cruz Biotechnology, Inc, Santa Cruz, CA. Each plasmid has Cas 9 ribonuclease and 20nt human OPG targeted guide RNA designed for maximum knockdown efficiency. Using Lipofectamine (Life Technologies #11668), SUM149PT and SUM1315MO2 cells were transfected with 1 μg of plasmid. The cells were also transfected with OPG HDR plasmid that has the puromycin selection casette. At 48h post-transfection, the transfected cells were selected with growth media supplemented with puromycin. Downregulation of OPG expression in each clone was screened by dot blot.

### Caspase assays

Caspase-9 and caspase-3/7 activity was measured using the caspase Glo assay (Promega G8211 and G8091) according to the manufacturer's instructions. In brief, SUM149PT and SUM1315MO2 cells were incubated with serum free F-12 media containing different concentrations of various inhibitors for 24h. After 24h, 100μl of the conditioned media was added to the proluminescent LEHD (caspase-9) or DEVD (caspase-3) substrate at a 1:1 ratio in a 200μl volume and incubated at room temperature for 1 h in the dark. Following incubation, luminescence was measured using a luminometer. All samples were assayed in triplicate. Luminescence was expressed as RLU and is proportional to the amount of caspase activity present in the sample.

### Statistical analysis

Three independent experiments were performed for each experiment to obtain reproducible results. The representative histograms are the average +/− SD of three independent experiments. The statistical significance of differences between experimental groups was determined by Student's t test. Statistical significance was calculated using GraphPad Prism 5 software.

## SUPPLEMENTARY MATERIALS FIGURE


