# Supplementary material for: Crosstalk between osteoprotegerin (OPG), fatty acid synthase (FASN) and, cycloxygenase-2 (COX-2) in breast cancer: implications in carcinogenesis
Source: Oncotarget. 2016 Jun 6;7(37):58953–74. doi: 10.18632/oncotarget.9835 (PMC5312288; doi:10.18632/oncotarget.9835)
Supplement: Supplementary file 1 [file oncotarget-07-58953-s001.pdf]

Crosstalk between osteoprotegerin (OPG), fatty acid synthase (FASN) and, cycloxygenase-2 (COX-2) in breast cancer: implications in carcinogenesis

SUPPLEMENTARY MATERIALS

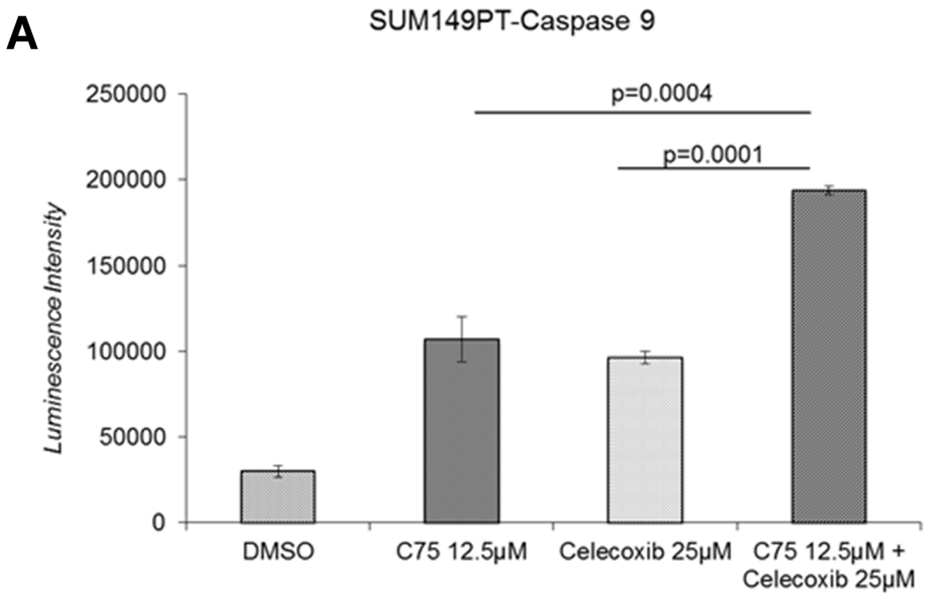

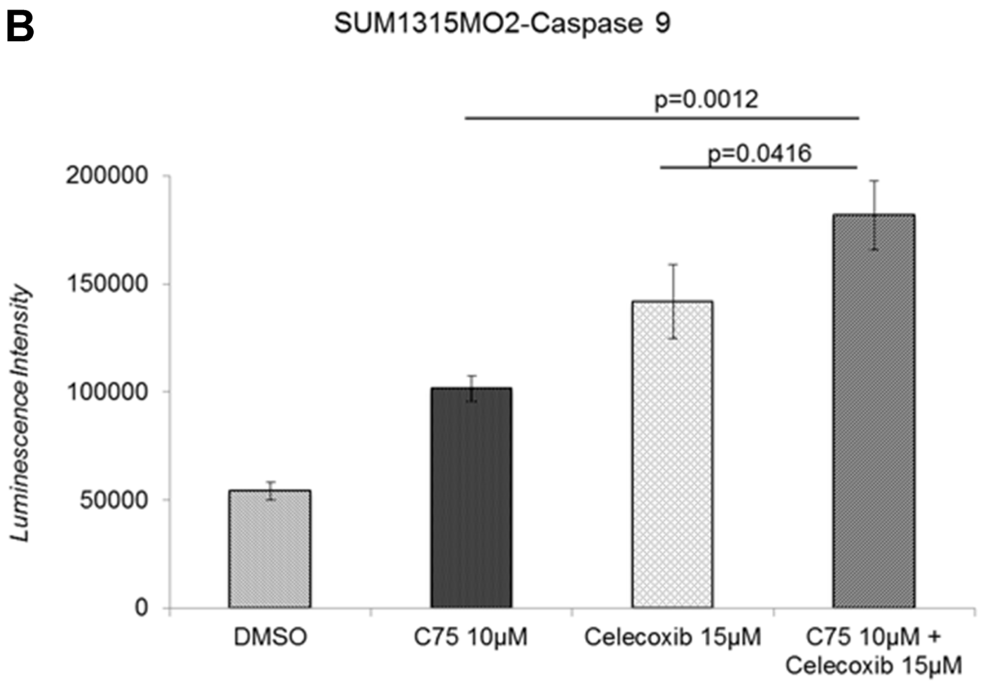

Supplementary Figure S1: Effect of the combined drug treatment on the caspase-9 pathway in aggressive breast cancer cells. A. and **B.** SUM149PT and SUM1315MO2 cells were plated in a 48-well plate. Upon confluency they were treated with respective C75 and celecoxib drug concentrations for 24h. After 24h, the cells were lysed in Caspase-9 respective buffer and luminescence was measured.
